# Study on the mechanism of miRNAs on liver injury in the condition of *Protoscocephalus alveolarus* transhepatic portal vein infection

**DOI:** 10.1002/iid3.1236

**Published:** 2024-04-23

**Authors:** Yazhou Zhu, Ming Li, Zihua Li, Jiahui Song, Wei Zhao

**Affiliations:** ^1^ Department of Pathogen Biology, School of Basic Medicine Ningxia Medical University Yinchuan China; ^2^ Ningxia Key Laboratory of Prevention and Control of Common Infectious Diseases Yinchuan China; ^3^ General Hospital of Ningxia Medical University Yinchuan China; ^4^ Department of Cell Biology and Genetics Ningxia Medical University Yinchuan China

**Keywords:** *Echinococcus multilocularis*, liver injury, miRNA

## Abstract

**Objective:**

To explore the role of miRNA in liver damage caused by *Echinococcus multilocularis* infection.

**Methods:**

Six female C57BL mice were randomly divided into two groups, the control group and the infection group. Mice in the control group were injected with 100 μL PBS through the hepatic portal vein, and mice in the infection group were infected with *E. multilocularis* via the hepatic portal vein to establish a mouse model of infection. Small RNA sequencing was performed for detecting the expression of miRNAs in the liver of mice infected with 2000 *E. multilocularis* after 3 months of infection, screen out miRNAs related to liver damage, and verify by RT‐PCR.

**Results:**

Seventy‐one differentially expressed miRNAs were found in the liver in comparison with control, and a total of 36 mouse miRNAs with |FC| >0.585 were screened out, respectively. In addition, Targetscan (V5.0) and miRanda (v3.3a) software were used to predict differential miRNAs target genes and functional enrichment of target genes. Functional annotation showed that “cytokine‐cytokine interaction,” “positive regulation of cytokine production,” “inflammatory response,” and “leukocyte activation” were enriched in the liver of *E. multilocularis*‐infected mice. Moreover, the pathways “human cytomegalovirus infection,” “cysteine and methionine metabolism,” “Notch signaling pathway,” and “ferroptosis” were involved in liver disease. Furthermore, four miRNAs (mmu‐miR‐30e‐3p, mmu‐miR‐203‐3p, mmu‐miR‐125b‐5p, and mmu‐miR‐30c‐2‐3p) related to liver injury were screened and verified.

**Conclusion:**

This study revealed that the expression profiling of miRNAs in the livers was changed after *E. multilocularis* infection, and improved our understanding of the transcriptomic landscape of hepatic echinococcosis in mice.

## Graphical abstract

miRNA is involved in the regulation of liver injury caused by *Echinococcus multilocularis* infection. mmu‐miR‐30e‐3p, mmu‐miR‐203‐3p, mmu‐miR‐125b‐5p, and mmu‐miR‐30c‐2‐3p may serve as potential diagnostic targets for liver injury.

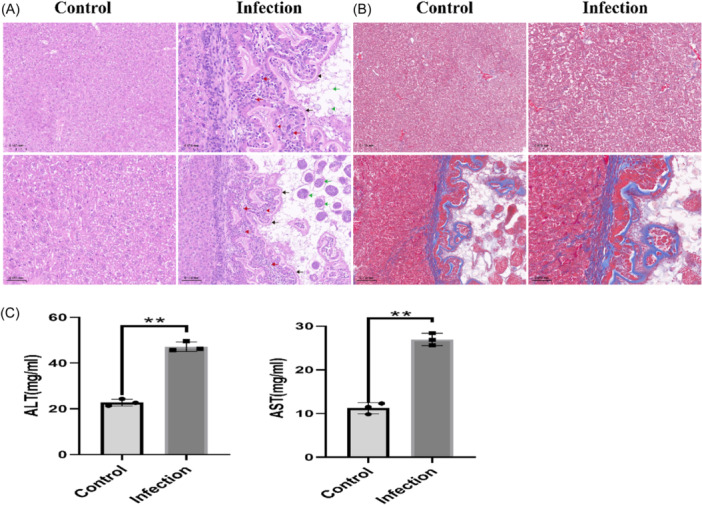

## 1 INTRODUCTION

Echinococcosis is a parasitic disease caused by the larvae of *Echinococcus* tapeworms parasitic on the human body.^1^, ^2^, ^3^ The disease is mainly distributed in the developed areas of animal husbandry in the western of China. Ningxia is also the main epidemic area of echinococcosis.^4^ Echinococcosis is mainly divided into two categories: cystic echinococcosis (CE) and alveolar echinococcosis (AE). The most common site of disease is mainly in the liver, and a few can also be found in the lungs and other organs.^5^, ^6^ The prevalence and incidence of CE is higher than AE.^7^, ^8^ But the damage of CE to the body is significantly higher than that of AE. CE is relatively easy to diagnose by B‐ultrasound imaging, and it can be removed by surgery.^9^, ^10^ AE showed infiltrative growth, and the early clinical symptoms were not obvious, so it is not easy to be discovered. Once discovered, it is often in the late stage and causes damage to organs. AE is very serious, which is also called “worm cancer.” The operation on AE is extremely difficult, which is necessary to resect the part of the liver (including pathogens) damaged by infiltration and growth. In the most severe cases, liver transplantation is required.^11^, ^12^, ^13^ However, the molecular mechanism of liver injury after *Echinococcus multilocularis* infection is still unclear.

MicroRNAs (miRNAs) are small noncoding RNAs with the capability of modulating gene expression at the posttranscriptional level.^14^, ^15^ In recent years, miRNAs have been widely involved in the regulation of cancer,^16^ infectious diseases,^17^ and other diseases.^18^ Altered expression of miRNAs is associated with liver metabolism dysregulation, liver injury, liver fibrosis, and tumor development, making miRNAs attractive therapeutic strategies for the diagnosis and treatment of liver diseases.^19^ Here, we injected *E. multiloculari*s through the hepatic portal vein to establish a mouse model of infection, after 3 months of infection, small RNA sequencing was performed, and miRNAs related to liver injury were screened out. These miRNAs may become the potential targets for the prevention and treatment of AE.

## 2 RESULTS

### 2.1 Establishment of a mouse model of hepatic portal vein infection

**Figure 1:**
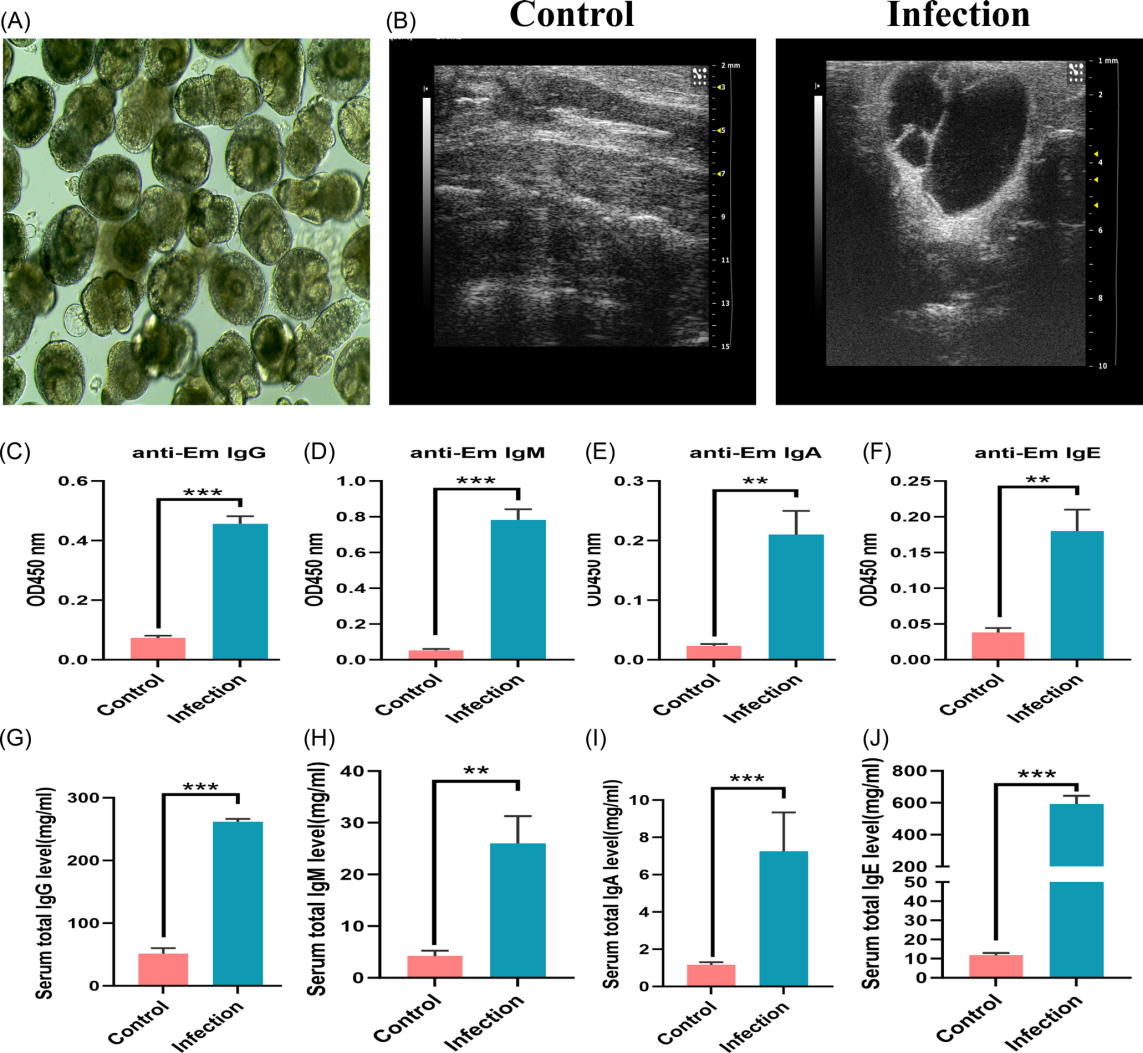
Identification of mouse models of infection. (A) Imaging of *Echinococcus multilocularis*. (B) B‐ultrasound detection of the liver in mice. (C−F) Changes of *E. multiloculari*s‐specific IgG, IgM, IgA, and IgE antibodies. (G−J) Changes of total IgG, IgM, IgA, and IgE antibodies. *p < .05, **p < .01, ***p < .005.

The cysts were removed from the gerbils, and the protoscoleiae were isolated (Figure 1A). One percent eosin staining was used to identify the viability of protoscolmus, and the viability exceeded 90%, which can be used for infection. A mouse model of secondary infection was established by injecting the protoscolmus via the hepatic portal vein. B‐ultrasound was used to detect the liver lesions of mice after 3 months of infection, as is shown in Figure 1B, there were obvious lesions in the liver tissue of the mice in the infection group, compared with the control group. The entry of pathogens into the body can induce the body to generate an immune response. So the *E. multilocularis*‐specific antibodies in the serum were detected by enzyme‐linked immunosorbent assay (ELISA). The results showed that the levels of *E. multilocularis*‐specific IgG, IgM, IgA, and IgE antibodies in the serum of mice in the infection group increased significantly (Figure 1C−F). And the total IgG, IgM, IgA, and IgE antibodies in serum were also significantly increased (Figure 1G−J). This suggested that the mouse model of infection through the hepatic portal vein was successfully constructed, and *E. multilocularis* infection can induce high levels of antibody responses.

### 2.2 *E. multilocularis* infection can cause liver injury

**Figure 2:**
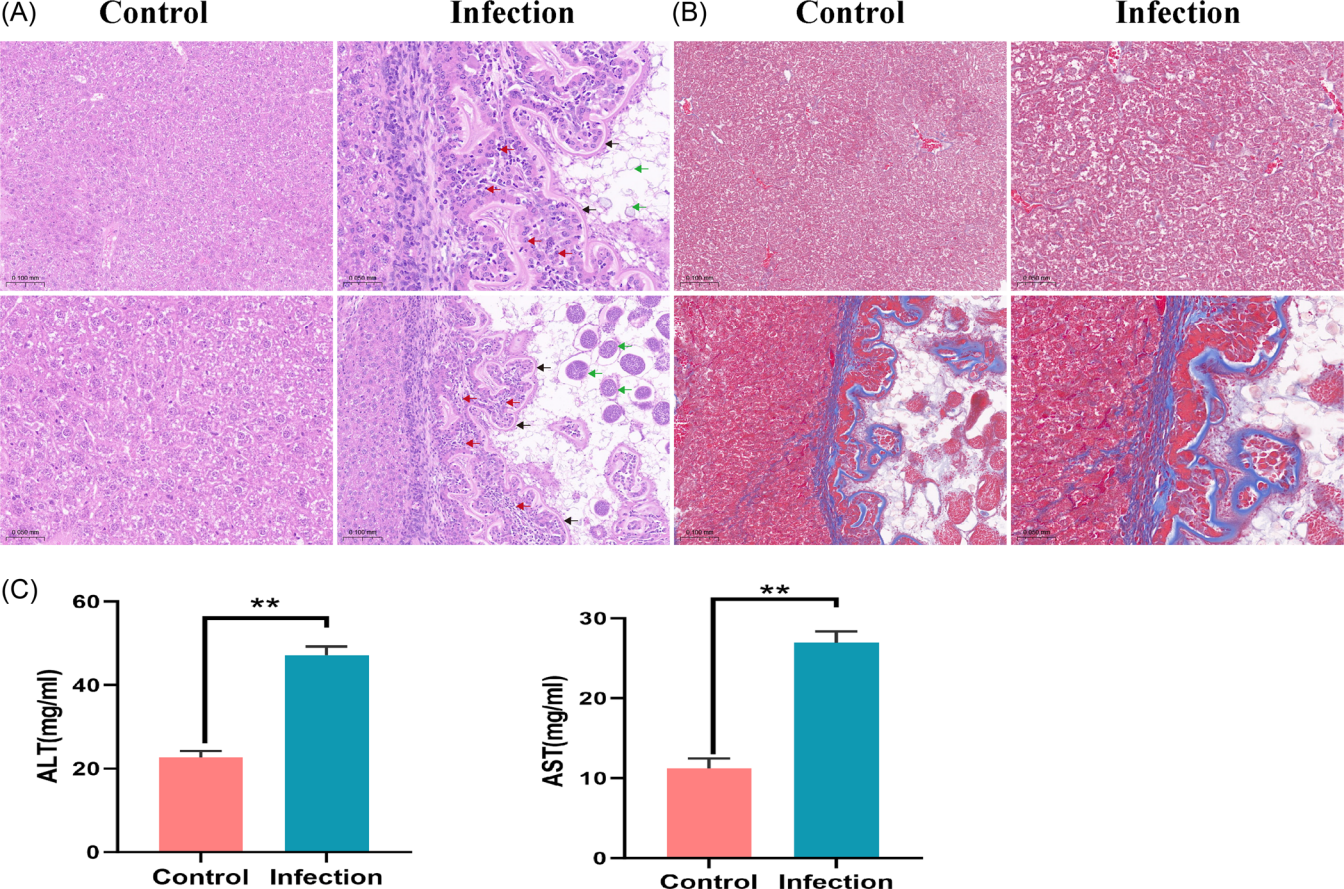
*Echinococcus multilocularis* infection causes liver damage. (A) The HE staining of mouse liver. (B) The Masson staining of mouse liver. (C) The level of AST and ALT in serum. *p < .05, **p < .01. The red arrows indicate inflammatory cells, black arrows indicate germinal layer, and green arrows indicate vesicle. HE, hematoxylin and eosin.

To determine whether *E. multiloculari*s infection can cause liver damage. we used the hematoxylin and eosin (HE) staining method to detect the liver pathological damage. The results showed that the mice in the infection group had obvious lesions in the liver and granulocytes gathered around the lesions compared with the control group (Figure 2A). The results of Masson staining showed that there was a large amount of collagen fiber deposition in the liver tissue of mice in the infection group (Figure 2B). In addition, the levels of AST and ALT in the serum of mice in the infection group were significantly higher than those in the control group (Figure 2C). This results suggested that *E. multiloculari*s parasitism can cause damage to liver tissue.

### 2.3 miRNAs differential expression

**Figure 3:**
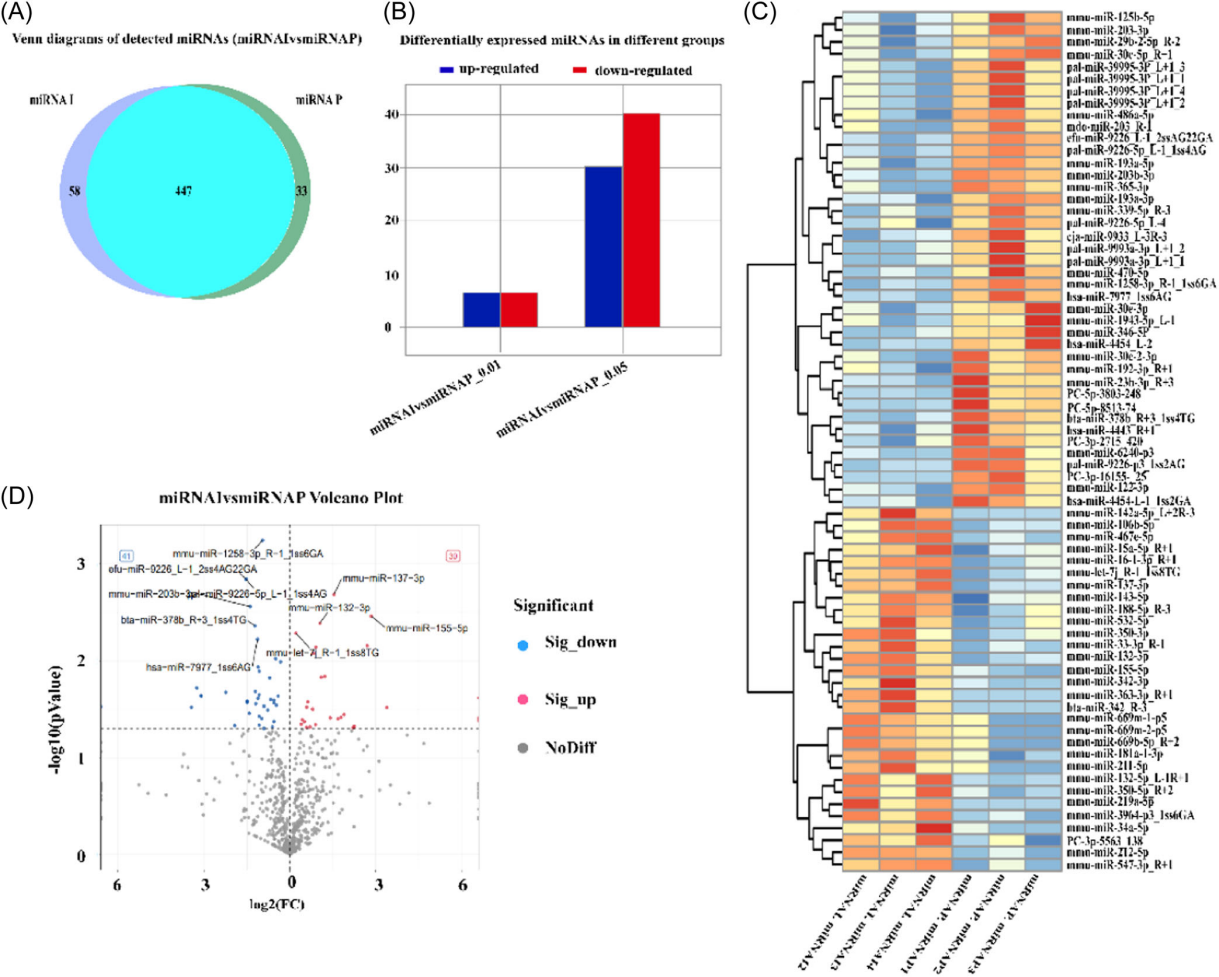
Differential expression of miRNAs. (A) Venn diagrams of detected miRNAs. (B) Differential expressed miRNAs in different groups. (C) Heat map of differential expression miRNAs. (D) Volcano plot of miRNAs.

*E. multiloculari*s infection can cause liver damage in mice, but the mechanism of damage is still unclear. This study mainly explores the mechanism of liver injury from the regulation of miRNAs. The mouse liver tissue close to the lesion site was isolated for small RNA sequencing. The raw data is shown in (Table 1), and a total of 71 differentially expressed miRNAs were screened out (Figure 3B−D, Supporting Information S1: Table 1), and deleted of non‐mouse miRNAs, there are 36 miRNAs among them with a large difference of |FC| >0.585 (Table 2), and these miRNAs may be related to liver damage caused by *E. multiloculari*s.

**Table 1:** The statistics of raw sequencing data for each library (mean ± standard deviation).

Sample	Total_reads	Total_bases	A%	T%	C%	G%	N%	Q20%	Q30%	GC%
miRNAI	15,188,065.67 ± 2,616,859.13	774,591,349.00 ± 133,459,836.10	23.12 ± 0.15	26.35 ± 0.44	19.92 ± 0.34	30.41 ± 0.24	0	98.19 ± 0.42	95.46 ± 0.94	50.33 ± 0.20
miRNAP	15,062,277.00 ± 3,795,412.81	768,176,127.00 ± 193,566,053.40	23.33 ± 0.25	26.28 ± 0.34	20.08 ± 0.27	30.31 ± 0.26	0	98.40 ± 0.13	95.55 ± 0.30	50.39 ± 0.09

**Table 2:** miRNA target gene prediction.

No	miRNA	mRNA	No	miRNA	mRNA	No	miRNA	mRNA
1	mmu‐miR‐467e‐5p	*Ciita*	13	mmu‐miR‐547‐3p_R + 1	*Arhgef2*	25	mmu‐miR‐350‐3p	*Ptgs2*
2	mmu‐miR‐155‐5p	*Il15ra*	14	mmu‐miR‐212‐5p	*Ms4a2*	26	mmu‐let‐7j_R‐1_1ss8TG	*Cd84*
3	mmu‐mir‐3964‐p3_1ss6GA	*Ccr1*	15	mmu‐miR‐132‐3p	*Ucp2*	27	mmu‐miR‐339‐5p_R‐3	*Il11Ra1*
4	mmu‐miR‐211‐5p	*Iigp1*	16	mmu‐miR‐33‐3p_R‐1	*Plekha1*	28	mmu‐miR‐23b‐3p_R + 3	*Akap12*
5	mmu‐mir‐669m‐2‐p5	*Mal*	17	mmu‐miR‐188‐5p_R + 1	*Ubash3a*	29	mmu‐miR‐30e‐3p	*Il1rn*
6	mmu‐miR‐669b‐5p_R + 2	*Mal*	18	mmu‐miR‐16‐1‐3p_R + 1	*Adam17*	30	mmu‐miR‐122‐3p	*Rhoh*
7	mmu‐miR‐669m‐1‐p5	*Mal*	19	mmu‐miR‐181a‐1‐3p	*Tnfsf13b*	31	mmu‐miR‐29b‐2‐5p_R‐2	*Ifngr1*
8	mmu‐miR‐34a‐5p	*Rhoh*	20	mmu‐miR‐106b‐5p	*Zfyve9*	32	mmu‐miR‐192‐3p_R + 1	*Gclm*
9	mmu‐miR‐142a‐5p_L + 2R‐3	*Ifngr1*	21	mmu‐miR‐15a‐5p_R + 1	*Ccr2*	33	mmu‐miR‐30c‐5p_R + 1	*Ifngr1*
10	mmu‐miR‐342‐3p	*Mefv*	22	mmu‐miR‐350‐5p_R + 2	*Fmr1*	34	mmu‐miR‐125b‐5p	*Was*
11	mmu‐miR‐137‐3p	*Rassf4*	23	mmu‐miR‐143‐5p	*Stmn1*	35	mmu‐miR‐30c‐2‐3p	*Prr51*
12	mmu‐miR‐132‐5p_L‐1R + 1	*Acs16*	24	mmu‐miR‐532‐5p	*Armc8*	36	mmu‐miR‐203‐3p	*Ctss*

### 2.4 miRNAs target gene prediction and functional enrichment

**Figure 4:**
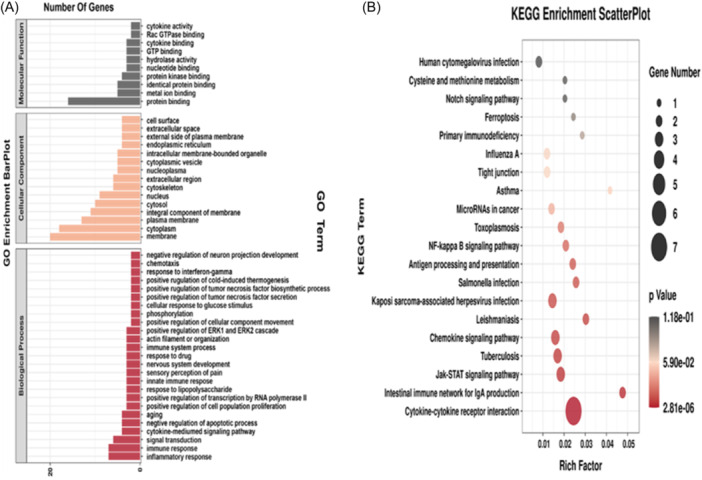
Functional enrichment of miRNA target genes. (A) GO enrichment barplot. (B) KEGG enrichment scatterplot. GO, gene ontology; KEGG, Kyoto encyclopedia of genes and genomes.

To elucidate the mechanism of differentially expressed miRNAs, we predicted the target genes of miRNAs using Targetscan (V5.0) and miRanda (v3.3a). In the prediction results, the mRNAs with the highest targeting score were selected as the target gene of miRNAs (Table 2). To find the potential biological associations of differential miRNAs, we performed gene ontology (GO) and Kyoto encyclopedia of genes and genomes (KEGG) pathway enrichment on these 36 mRNAs. The GO term is shown in Figure 4A, GO enrichment results showed that differentially expressed miRNAs target genes were mainly involved in “inflammatory response,” “immune response,” “signal transduction,” “cytokine‐mediumed signaling pathway,” “negative regulation of the apoptotic process,” and “aging” biological process. And these mRNAs may participate in the formation of membrane, cytoplasm, plasma membrane, cytosol, and nucleus. KEGG enrichment analysis showed that pathways mainly included “cysteine and methionine metabolism,” “Notch signaling pathway,” “ferroptosis,” and “antigen processing and presentation” in the *E. multilocularis* infection (Figure 4B). These results showed that *E. multiloculari*s infection can affect liver metabolic function and changes in signaling pathways.

### 2.5 Screening of miRNAs related to liver injury

*E. multiloculari*s enters the liver tissue along with the blood circulation, which induces an immune response in the liver and recruits a large number of inflammatory cells that release proinflammatory cytokines and promote the proliferation of fibroblasts. With the expansion of the lesion, immune tolerance appears in the liver, the expression of anti‐inflammatory cytokines increases, the imbalance between fibrosis formation and degradation aggravates the degree of liver fibrosis, and the expression of collagen increases. Therefore, we screened four miRNAs related to liver injury (inflammatory response, immune response, fibrosis process) (Table 3). mmu‐miR‐30e‐3p may target IL‐1rn mRNA to participate in the inflammatory response. mmu‐miR‐125b‐5p may target was mRNA to regulate the immune response. mmu‐miR‐30c‐2‐3p may target the prr5l gene to regulate the fibroblast migration. And mmu‐miR‐203‐3p may target ctss mRNA to regulate the immune response.

**Table 3:** Liver injury‐related miRNA expression.

Number	miRNA name	miRNA sequence	mRNA	Target score
1	mmu‐miR‐30e‐3p	CTTTCAGTCGGATGTTTACAC	*Il‐1rn*	96
2	mmu‐miR‐203‐3p	GTGAAATGTTTAGGACCACTAG	*Ctss*	99
3	mmu‐miR‐125b‐5p	TCCCTGAGACCCTAACTTGTA	*Was*	99
4	mmu‐miR‐30c‐2‐3p	CTGGGAGAAGGCTGTTTACTCT	*Prr5l*	97

### 2.6 Verification of differentially expressed miRNAs

**Figure 5:**
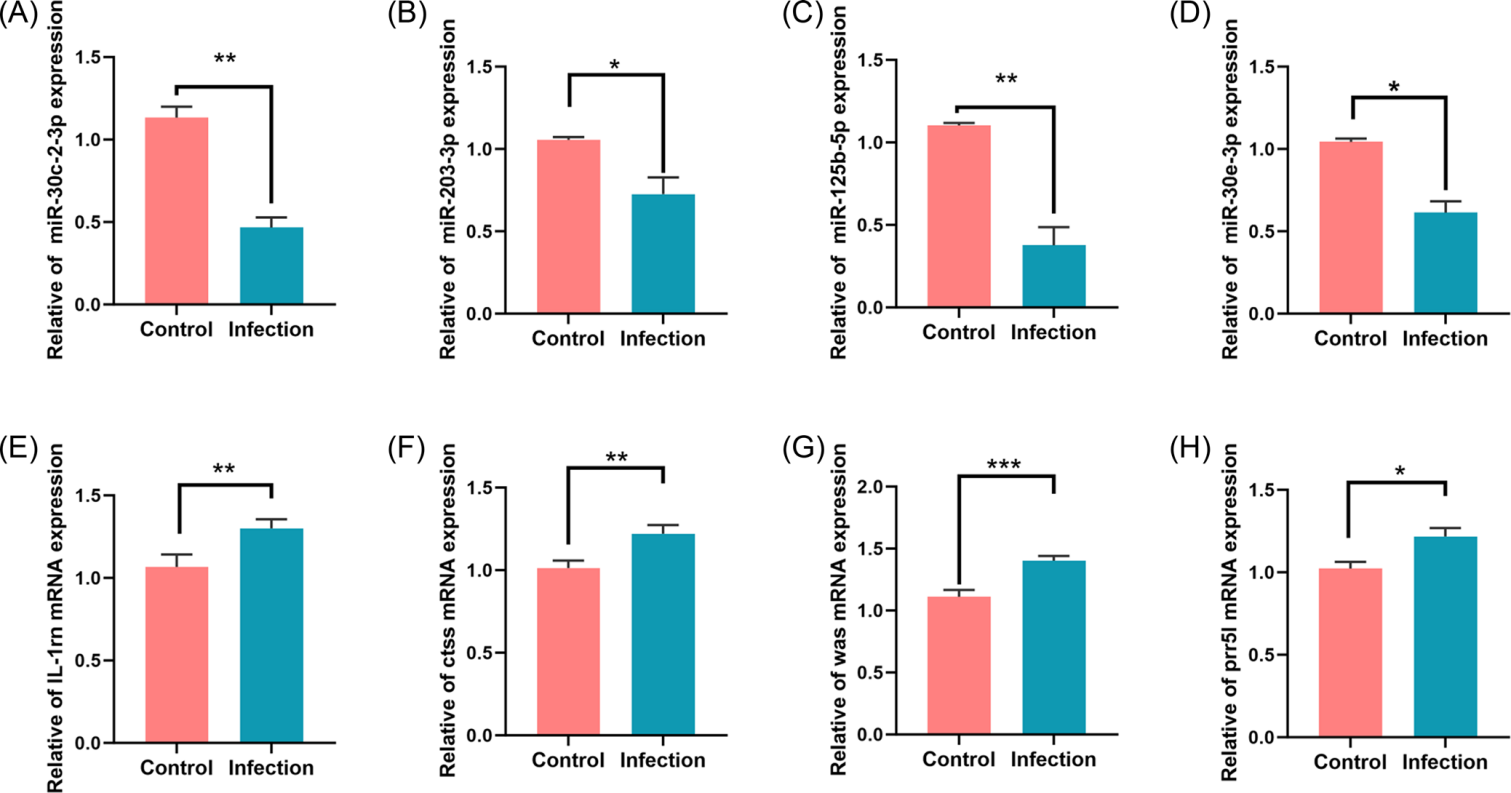
Validation of liver injury‐related miRNAs and target genes by RT‐qPCR. (A) Change of log2 (fold change) and fold change of differential expression gene. (B) The level of liver injury‐related miRNAs expression. (C) The level of mRNA expression. *p < .05, **p < .01, ***p < .001.

To evaluate the reliability of the sequencing results, the screened miRNAs and mRNAs were verified by RT‐qPCR. The results showed that compared with the control group, the expression of mmu‐miR‐30e‐3p, mmu‐miR‐203‐3p, mmu‐miR‐125b‐5p, and mmu‐miR‐30c‐2‐3p in the liver of infected mice were significantly reduced, and the expression levels of IL‐1rn, Ctss, Was, and Prr5l were significantly increased (Figure 5B,C). The RT‐qPCR results are consistent with the sequencing results.

## 3 DISCUSSION

AE is a zoonotic parasitic disease that seriously endangers human health.^20^ The study of *E. multilocularis* has always lacked stable experimental animal models. The traditional infection models are secondary infection mouse models constructed by intraperitoneal injection and liver puncture.^21^, ^22^ Although it can reflect that the body is infected by *E. multilocularis* to a certain extent, the infection mode is far from the natural infection. During natural infection, humans become infected when they inadvertently ingest soil, water, or food contaminated with Echinococcus eggs excreted in dog feces. The eggs germinate in the small intestine to form hexacarcinoma, which can adhere and penetrate the intestinal wall, enter blood vessels, and reach and parasitize in the liver tissue along with the blood circulation.^23^, ^24^ In recent years, Abuduaini Abulizi and his colleagues established a novel mouse model of infection by injecting *E. multilocularis* through the hepatic portal vein.^25^ This model can more accurately simulate the natural infection process.

In this study, the infection mouse model was constructed by injecting *E. multiloculari*s through the hepatic portal vein. After 3 months of infection, B‐ultrasound results found that the liver of the mice in the infection group had obvious lesions and *E. multiloculari*s antigen‐specific antibodies in serum significantly increased, which indicates that the infection mouse model was successfully constructed. The results of HE and Masson staining showed that the infection of *E. multiloculari*s could cause liver damage, and a large number of collagen fibers were formed. In addition, the level of ALT and AST in serum were increased. These results suggested that *E. multiloculari*s infection can cause liver tissue damage. However, the mechanism of liver injury is still unclear. Thus, the present study explored the mechanism of liver injury from the perspective of miRNA by using the RNA‐seq technique. A total of 71 differential expression miRNAs were obtained, and a total of 36 mouse miRNAs with |FC| >0.585 were screened out. Target gene prediction and functional enrichment of target genes were carried out on the 36 miRNAs screened out, the results showed that target genes were involved in many biological processes, such as “inflammatory response,” “immune response,” and “signal transduction.” KEGG analysis showed that a series of pathways (“Notch signaling pathway,” “ferroptosis,” “cysteine and methionine metabolism”) were involved in liver disease, indicating that *E. multiloculari*s induced the liver immunity reaction of hosts and activated signaling pathways related to immune response, such as Notch signaling pathway.

*E. multiloculari*s enters the liver tissue along with the blood circulation, the liver tissue will also generate an immune response to resist infection and recruit a large number of inflammatory cells at the same time to release inflammatory cytokines, and finally play an anti‐infection immune process. With the expansion of the parasite infection focus, the unbalanced between formation and degradation of fibrosis will aggravate the degree of liver fibrosis. Therefore, this study focuses on three aspects (inflammatory response, immune response, and fibrosis formation) to search for miRNAs related to liver injury. In this study, we screened a total of four miRNAs and four miRNA target genes, which were mmu‐miR‐30e‐3p and IL‐1rn, mmu‐miR‐203‐3p and ctss, mmu‐miR‐125b‐5p and was, mmu‐miR‐30c‐2‐3p and prr5l. Wei Cong et al. suggested that mmu‐miR‐30e‐3p were also differentially expressed in liver tissue from rats with acute *Toxoplasma gondii* infection,^26^ however, mmu‐miR‐30e‐3p targeting IL‐1rn regulates the inflammatory response caused by parasitic infections and no studies have been reported. Hu et al. found that miR‐203‐3p may bind to vascular endothelial growth factor A (Vegfa) and participate in snhg8‐mediated apoptosis and angiogenesis in AML cells.^27^ Moreover, mmu‐miR‐203‐3p was identified as a potential diagnostic biomarker and indicator of Sjögren's syndrome.^28^ However, there are no studies have been reported on mmu‐miR‐203‐3p in liver injury caused by parasitic infections. mmu‐miR‐125b‐5p is extensively involved in biological processes such as tua protein phosphorylation, cerebral ischemic tolerance, and neuroregeneration.^29^ In recent years, it has also been reported that mmu‐miR‐125b‐5p was found to be downregulated in the peripheral blood of *Trichinella*‐infected mice.^30^ None of the four liver injury‐associated miRNAs screened in this study have been studied in models of liver injury, and these miRNAs may serve as potential diagnostic targets for AE.

## 4 MATERIALS AND METHODS

### 4.1 Animal

Female C57BL mice aged 6−8 weeks were purchased from the Experimental Animal Center of Ningxia Medical University. All mice were kept in a temperature‐controlled, light‐cycle room in animal facilities under specific pathogen‐free conditions according to the national guidelines for animal care and use, with food and water ad libitum.

### 4.2 Isolation of protozoan and vitality identification

Protoscocras (PSCs) were obtained from *E. multilocularis*‐infected gerbils. In brief, *E. multilocularis* infected cyst tissue was isolated from gerbils and stripped components of the mouse. PSCs were isolated immediately by pushing the cyst through a 300‐mesh strainer in PBS buffer, and collected filtrate. The filtrate was naturally settlemented for 5 min, discard the supernatant, and washed two times with PBS. PSCs were stained with 1% eosin (Beyotime) to test vitality. If the vitality was over 95%, the PSCs could inject in mice.

### 4.3 Construction of infection mouse model

Six female C57 mice were randomly divided into a control group and an infection group, with three mice in each group. The infection mouse model was established by injecting PSCs via the hepatic portal vein. In brief, the mice in the infection group were injected with PSCs via the portal vein, and portal saline injection was defined as the control group. A middle abdominal incision was made in mice under anesthetic, and 2000 PSCs/200 μL sediment or saline was injected via portal vein by using a 0.45 × 15RWLB venous infusion needle. After injection, a cotton bud was pressed on the puncture site for 5 min to provide hemostasis and to prevent intraperitoneal spillage of the PSCs. The abdominal cavity was then closed and then the mice were put on the warm stage to promote waking.

### 4.4 RNA extraction

Liver tissue RNA was extracted by the Trizol method. In brief, approximately 50 mg of liver tissue was first placed in a mortar (Thermo Fisher Scientific), ground into a powder with liquid nitrogen, and transferred to an enzyme‐free tube, and 1 mL TRIZOL (Invitrogen) reagent was added to the homogenization to lyse sample for 5 min at room temperature in the dark. Then, 200 μL of chloroform per was added, and shake tubes vigorously by hand for 15 s. The sample was allowed to stand at room temperature for 3 min, and centrifuged at 12,000*g* for 10 min at 4°C. Pipette the supernatant into another new enzyme‐free tube, 400 μL Isopropanol was added, and shake tubes slightly by hand for 15 s. The sample was allowed to stand at room temperature for 3 min, and centrifuged at 12,000*g* for 10 min at 4°C. Discard the supernatant, add 1 mL 75% ethanol into the tube, and shake the tube for 30 s. Centrifuged at 7500*g* for 5 min at 4°C. Then, discard the supernatant and let the alcohol evaporate naturally. Finally, the RNA pellet was redissolved with the 20 μL enzyme‐free water. The RNA degradation and contamination were detected with a 1% agarose gel test, and RNA concentration was detected by NanoDrop Microvolume Spectrophotometers (Thermo Fisher Scientific).

### 4.5 Small RNA library construction, sequence analysis, and identification of miRNAs

Three mouse liver RNAs of each group were used for small RNA sequencing. The experimental procedure mainly includes two parts: preparation of the library and sequencing experiment. Small RNA sequencing library preparation using TruSeq Small RNA Sample Prep Kits (Illumina). After library preparation, the libraries were sequenced using Illumina Hiseq. 2000/2500 with a single‐end 1 × 50 bp read length.

The miRNAs data analysis software is the self‐developed ACGT101‐miR (LC Sciences). The analysis flow of the software is shown in Figure 1B. In brief, remove 3’ connectors and rubbish sequences to get clean data and screening of sequences with base length between 18 and 26 nt. Then, the remaining sequences were compared to (without miRNA) mRNA, RFam, and Repbase databases and filtered and obtaining valid data and comparing precursors and genomes for miRNAs identification. The miRNA identification base is linked to the miRBase and the genome of the species, and the degree of accuracy is highly correlated with the completeness of the database. Clean Data was used to identify small RNAs and calculate the miRNAs expression levels identified in each sample using ACGT101‐miR. Expression was counted and used to assess the correlation of gene expression characteristics and differentially expressed miRNAs within and between groups of samples.

### 4.6 Target prediction of the differential miRNAs

Differential miRNAs target gene prediction was performed using both software A and B, and target genes predicted by both software were screened according to scoring criteria. Target genes with context score percentages less than 50 are removed in the targetscan algorithm. Target genes with maximum free energy (Max energy) greater than −10 are removed in the miRanda algorithm (i.e., the threshold is TargetScan score ≥50, miranda_Energy <−10). Finally, the intersection of these two software was taken as the final target gene of the differential miRNAs.

### 4.7 Functional annotation of the differential miRNAs

To analyze the biological function of differential miRNAs, the predicted miRNAs target genes were annotated using GO enrichment (ftp://ftp.ncbi.nih.gov/gene/DATA/gene2go.gz) and KEGG pathway analysis (http://www.genome.jp/kegg). The KEGG analysis provides information on the signal transduction and disease pathways of target genes. This information provides a basis for studying the function and involvement of differential miRNAs in pathways. GO is an internationally standardized gene function classification system. It includes three ontologies that describe the molecular functions of genes, cellular components, and the biological processes involved.

### 4.8 HE

HE staining (Servicebio) was performed on thin slices that were embedded in a wax block of liver tissue. The whole dyeing process includes five contents: dewaxing, dyeing, dehydration, transparent, and mounting. The overall characteristics of the tissue were observed under a low‐power microscope, and then the images of representative areas were observed and collected.

### 4.9 Detection of ALT and AST in serum

The contents of AST and ALT in mouse serum were detected by an automatic biochemical analyzer (Thermo Fisher Scientific).

### 4.10 Quantitative real‐time PCR analysis

Following the manufacturer's instructions, total RNA was isolated from the mouse liver using Trizol reagent (Invitrogen). And then reverse‐transcribed into cDNA using the qPCR RT kit (GeneCopoeia). The cDNA was analyzed using real‐time qPCR with a StepOnePlusTM Real‐Time PCR System (Thermo Fisher Scientific). Each reaction was performed in triplicate in a 96‐well plate. The expression of each gene was normalized by the expression of GAPDH. miRNA and mRNA primers are listed in Table 4.

**Table 4:** The sequences of miRNA and mRNA primers.

No	miRNAs/mRNAs	Forward primer	Reverse primer
1	mmu‐miR‐30e‐3p	GGCTTTCAGTCGGATGTTTACAGC	GCGGTCGGACTACATCAT
2	mmu‐miR‐203‐3p	CGCGTGAAATGTTTAGGACCACTAG	GCGGTCGGACTACATCAT
3	mmu‐miR‐125b‐5p	TCCCTGAGACCCTAACTTGTGA	GCGGTCGGACTACATCAT
4	mmu‐miR‐30c‐2‐3p	CTGGGAGAAGGCTGTTTACTCT	GCGGTCGGACTACATCAT
5	*IL‐1rn*	CCCACCACCAGCTTTGAGTCAG	GGACGGTCAGCCTCTAGTGTTG
6	*Was*	CGAGGATGAAGAGGATGATGAATGG	ATGGCAGAGTGAGTGTGGAGAG
7	*Prr5l*	TGGTGAAGCAAGTGGTGTCTCC	CCGTGGTCATCAGAGAGGCATAG
8	*Ctss*	TTGGGGCCTTAACTTTGGTGATC	GCAATAACTAGCAATTCCGCAGTG
9	*U6*	CTTCGGCAGCACATATACTAAAAT	CGCTTCACGAATTTGCGTGTCAT
10	*GAPDH*	AGAAGGCTGGGGCTCATTTG	AGGGGCCATCCACAGTCTTC

### 4.11 Antibody testing in serum

According to the requirements of the reagent instructions, the antibodies in the serum were detected by ELISA. In brief, 10 μg/mL *E. multilocularis* crude antigen was coated on the ELISA plate and placed at 4°C overnight. The plates were washed five times with PBST (0.05% Tween 20 PBS) and blocked with 5% skim milk powder in PBST at 37°C for 1 h. After washing five times with PBST, the plates were incubated with mouse serum (1:500) in 5% skim milk powder in PBST for 2 h and washed five times with PBST for 3 min. And 100 μL each of horseradish peroxidase‐conjugated anti‐mouse IgM, IgG, IgA (Abcam), and IgE (Invitrogen) were added to enzyme plates and incubated at 37°C for 1 h. After washing, 100 μL TMB Single‐Component Substrate solution (Solarbio) was added for 8−10 min, and the reaction was stopped by 2M H_2_SO_4_. The absorbance was measured at 450 nm using an ELISA reader (Thermo Fisher Scientific).

### 4.12 Statistical analysis

Differential miRNAs expression levels were analyzed by one‐way ANOVA test. For the sample data analysis of this experiment, the differential expression genes were screened by *p* < .05 as the threshold. The differentially expressed genes are defined as those with fold change ≥1 (upregulated) or <1 (downregulated) between the groups.

## 5 CONCLUSION

miRNA is involved in the regulation of liver injury caused by *E. multilocularis* infection, and mmu‐miR‐30e‐3p, mmu‐miR‐203‐3p, mmu‐miR‐125b‐5p, and mmu‐miR‐30c‐2‐3p may serve as potential diagnostic targets for liver injury.

## AUTHOR CONTRIBUTIONS

Wei Zhao was in charge of the project design. Ming Li and Zihua Li were responsible for model construction and RNA extraction. Jiahui Song and Yazhou Zhu were responsible for analyzing data and RT‐PCR experiments.

## CONFLICT OF INTEREST STATEMENT

The authors declare no conflict of interest.

## ETHICS STATEMENT

This study was conducted under the rules of the Guide for the Care and Use of Laboratory Animals and the Institutional Animal Use and Care Committee of Ningxia Medical University (approval number: KYLL‐2021‐765).

## Supporting information

Supporting information.

## Data Availability

All data in this experiment are true and valid.
