# Supplementary material for: Study on the mechanism of miRNAs on liver injury in the condition of Protoscocephalus alveolarus transhepatic portal vein infection
Source: Immun Inflamm Dis. 2024 Apr 23;12(4):e1236. doi: 10.1002/iid3.1236 (PMC11037255; doi:10.1002/iid3.1236)
Supplement: Supplementary file 1 — Supporting information. [file IID3-12-e1236-s001.docx]

Table S1 differentially expressed miRNAs of infection

| Index | miR_name | miR_seq | up/down | Fc | log2fc | pvalue |
| --- | --- | --- | --- | --- | --- | --- |
| 1 | mmu-miR-1258-3p_R-1_1ss6GA | TTAGGAAATTAGCTCAGCAGT | down | 0.52 | -0.95 | 5.72E-04 |
| 2 | efu-miR-9226_L-1_2ss4AG22GA | CAGGTCCCTGTTCGGGCGCCA | down | 0.35 | -1.52 | 1.45E-03 |
| 3 | pal-miR-9226-5p_L-1_1ss4AG | CAGGTCCCTGTTCGGGCGCCA | down | 0.35 | -1.52 | 1.45E-03 |
| 4 | mmu-miR-137-3p | TTATTGCTTAAGAATACGCGTAG | up | 2.91 | 1.54 | 2.07E-03 |
| 5 | mmu-miR-203b-3p | TTGAACTGTCAAGAACCACTGG | down | 0.38 | -1.38 | 2.76E-03 |
| 6 | mmu-miR-155-5p | TTAATGCTAATTGTGATAGGGGT | up | 7.17 | 2.84 | 3.47E-03 |
| 7 | mmu-miR-132-3p | TAACAGTCTACAGCCATGGTCG | up | 2.07 | 1.05 | 4.13E-03 |
| 8 | bta-miR-378b_R+3_1ss4TG | ACTGGACTTGGAGTCAGAAGGCTTA | down | 0.43 | -1.22 | 4.38E-03 |
| 9 | mmu-let-7j_R-1_1ss8TG | TGAGGTAGTAGTTTGTGCTGTTA | up | 1.16 | 0.21 | 5.23E-03 |
| 10 | hsa-miR-7977_1ss6AG | TTCCCGGCCAACGCACCA | down | 0.46 | -1.12 | 6.02E-03 |
| 11 | mmu-mir-3964-p3_1ss6GA | CATAAAGTAGAAAGCACTAA | up | 6.51 | 2.70 | 7.01E-03 |
| 12 | mmu-miR-33-3p_R-1 | CAATGTTTCCACAGTGCATCA | up | 1.87 | 0.90 | 7.28E-03 |
| 13 | mmu-miR-16-1-3p_R+1 | CCAGTATTGACTGTGCTGCTGAA | up | 1.77 | 0.82 | 8.39E-03 |
| 14 | mmu-miR-122-3p | AAACGCCATTATCACACTAAAT | down | 0.71 | -0.50 | 9.51E-03 |
| 15 | mmu-miR-339-5p_R-3 | TCCCTGTCCTCCAGGAGCTC | down | 0.80 | -0.33 | 1.03E-02 |
| 16 | mmu-miR-193a-3p | AACTGGCCTACAAAGTCCCAGT | down | 0.46 | -1.11 | 1.16E-02 |
| 17 | mmu-miR-365-3p | TAATGCCCCTAAAAATCCTTAT | down | 0.48 | -1.07 | 1.28E-02 |
| 18 | mmu-miR-547-3p_R+1 | CTTGGTACATCTTTGAGTGAGT | up | 2.33 | 1.22 | 1.46E-02 |
| 19 | mmu-miR-212-5p | ACCTTGGCTCTAGACTGCTTACT | up | 2.14 | 1.10 | 1.48E-02 |
| 20 | mmu-miR-30c-2-3p | CTGGGAGAAGGCTGTTTACTCT | down | 0.61 | -0.71 | 1.51E-02 |
| 21 | mmu-miR-346-5p | TGTCTGCCCGAGTGCCTGCCTCT | down | 0.11 | -3.25 | 1.91E-02 |
| 22 | hsa-miR-4454_L-1_1ss2GA | AATCCGAGTCACGGCACCA | down | 0.43 | -1.21 | 2.09E-02 |
| 23 | cja-miR-9933_L-3R-3 | TATCACCCATGACTGATG | down | 0.21 | -2.23 | 2.12E-02 |
| 24 | PC-3p-2715_420 | TTGAACTGTCAAGAACCACTGGT | down | 0.48 | -1.07 | 2.25E-02 |
| 25 | pal-miR-9993a-3p_L+1_2 | CATCTCGGTGGGACCTCCA | down | 0.12 | -3.10 | 2.31E-02 |
| 26 | pal-miR-9993a-3p_L+1_1 | AATCTCGGTGGGACCTCCA | down | 0.12 | -3.10 | 2.31E-02 |
| 27 | mmu-miR-23b-3p_R+3 | ATCACATTGCCAGGGATTACCACT | down | 0.75 | -0.41 | 2.32E-02 |
| 28 | bta-miR-342_R-3 | TCTCACACAGAAATCGCACCCA | up | inf | inf | 2.42E-02 |
| 29 | hsa-miR-4454_L-2 | ATCCGAGTCACGGCACCA | down | 0.46 | -1.11 | 2.44E-02 |
| 30 | mmu-miR-125b-5p | TCCCTGAGACCCTAACTTGTGA | down | 0.63 | -0.67 | 2.52E-02 |
| 31 | mmu-miR-30c-5p_R+1 | TGTAAACATCCTACACTCTCAGCT | down | 0.68 | -0.56 | 2.56E-02 |
| 32 | pal-miR-9995-3p_L+1_1 | AATCTCGGTGGAACCTCCA | down | 0.36 | -1.48 | 2.63E-02 |
| 33 | pal-miR-9995-3p_L+1_2 | CATCTCGGTGGAACCTCCA | down | 0.36 | -1.48 | 2.63E-02 |
| 34 | pal-miR-9995-3p_L+1_4 | TATCTCGGTGGAACCTCCA | down | 0.36 | -1.48 | 2.63E-02 |
| 35 | pal-miR-9995-3p_L+1_3 | GATCTCGGTGGAACCTCCA | down | 0.36 | -1.48 | 2.63E-02 |
| 36 | mmu-miR-15a-5p_R+1 | TAGCAGCACATAATGGTTTGTGT | up | 1.55 | 0.63 | 2.66E-02 |
| 37 | hsa-miR-4443_R+1 | TTGGAGGCGTGGGTTTTT | down | 0.36 | -1.49 | 2.69E-02 |
| 38 | mmu-miR-29b-2-5p_R-2 | CTGGTTTCACATGGTGGCTTAGA | down | 0.68 | -0.55 | 2.73E-02 |
| 39 | mmu-miR-193a-5p | TGGGTCTTTGCGGGCAAGATGA | down | 0.44 | -1.19 | 2.77E-02 |
| 40 | mmu-miR-30e-3p | CTTTCAGTCGGATGTTTACAGC | down | 0.72 | -0.47 | 2.89E-02 |
| 41 | PC-5p-3803_248 | TGGAGTGACAATGGTGTTT | down | 0.53 | -0.91 | 2.95E-02 |
| 42 | PC-3p-16155_25 | GTTCTTAGTTGGTGGAGCG | down | -inf | -inf | 2.98E-02 |
| 43 | mmu-miR-350-5p_R+2 | AAAGTGCATGCGCTTTGGGAC | up | 1.50 | 0.59 | 3.01E-02 |
| 44 | mmu-miR-470-5p | TTCTTGGACTGGCACTGGTGAGT | down | 0.09 | -3.43 | 3.02E-02 |
| 45 | mmu-miR-467e-5p | ATAAGTGTGAGCATGTATATGT | up | 10.43 | 3.38 | 3.04E-02 |
| 46 | mmu-miR-181a-1-3p | ACCATCGACCGTTGATTGTACC | up | 1.73 | 0.79 | 3.16E-02 |
| 47 | mmu-miR-203-3p | GTGAAATGTTTAGGACCACTAG | down | 0.59 | -0.77 | 3.23E-02 |
| 48 | PC-5p-8513_74 | AGTCTTACAGTCCTTACCA | down | 0.37 | -1.42 | 3.49E-02 |
| 49 | mmu-miR-34a-5p | TGGCAGTGTCTTAGCTGGTTGT | up | 3.71 | 1.89 | 3.59E-02 |
| 50 | pal-mir-9226-p3_1ss2AG | AGGTCCCTGTTCGGGCGCCA | down | 0.48 | -1.07 | 3.75E-02 |
| 51 | mmu-miR-142a-5p_L+2R-3 | CCCATAAAGTAGAAAGCACT | up | 3.45 | 1.79 | 3.86E-02 |
| 52 | mmu-miR-219a-5p | TGATTGTCCAAACGCAATTCT | up | inf | inf | 3.88E-02 |
| 53 | mmu-miR-132-5p_L-1R+1 | ACCGTGGCTTTCGATTGTTACT | up | 2.65 | 1.41 | 3.89E-02 |
| 54 | mdo-miR-203_R-1 | GTGAAATGTTTAGGACCACTT | down | 0.51 | -0.98 | 3.94E-02 |
| 55 | mmu-miR-342-3p | TCTCACACAGAAATCGCACCCGT | up | 3.20 | 1.68 | 3.98E-02 |
| 56 | mmu-miR-532-5p | CATGCCTTGAGTGTAGGACCGT | up | 1.35 | 0.43 | 4.08E-02 |
| 57 | mmu-miR-363-3p_R+1 | AATTGCACGGTATCCATCTGTAT | up | inf | inf | 4.15E-02 |
| 58 | mmu-miR-192-3p_R+1 | CTGCCAATTCCATAGGTCACAGT | down | 0.68 | -0.55 | 4.24E-02 |
| 59 | mmu-miR-143-5p | GGTGCAGTGCTGCATCTCTGG | up | 1.42 | 0.50 | 4.25E-02 |
| 60 | mmu-miR-350-3p | TTCACAAAGCCCATACACTTTC | up | 1.31 | 0.39 | 4.52E-02 |
| 61 | mmu-miR-188-5p_R+1 | CATCCCTTGCATGGTGGAGGGT | up | 1.86 | 0.89 | 4.57E-02 |
| 62 | mmu-miR-1943-5p_L-1 | AGGGAGGATCTGGGCACCTGGA | down | 0.48 | -1.07 | 4.63E-02 |
| 63 | mmu-mir-6240-p3 | ATTTCTGCCCAGTGCTCTGAA | down | 0.26 | -1.93 | 4.67E-02 |
| 64 | mmu-miR-211-5p | TTCCCTTTGTCATCCTTTGCCT | up | 4.75 | 2.25 | 4.77E-02 |
| 65 | mmu-miR-106b-5p | TAAAGTGCTGACAGTGCAGAT | up | 1.63 | 0.71 | 4.82E-02 |
| 66 | pal-miR-9226-5p_L-4 | GTCCCTGTTCGGGCGCCA | down | 0.66 | -0.61 | 4.82E-02 |
| 67 | PC-3p-5563_138 | ATCACATTGCCAGGGATTTTT | up | 1.50 | 0.58 | 4.88E-02 |
| 68 | mmu-mir-669m-2-p5 | AGTTTTGTGTGCATGTGCATGTGT | up | 4.64 | 2.22 | 4.90E-02 |
| 69 | mmu-miR-669b-5p_R+2 | AGTTTTGTGTGCATGTGCATGTGT | up | 4.64 | 2.22 | 4.90E-02 |
| 70 | mmu-mir-669m-1-p5 | AGTTTTGTGTGCATGTGCATGTGT | up | 4.64 | 2.22 | 4.90E-02 |
| 71 | mmu-miR-486a-5p | TCCTGTACTGAGCTGCCCCGAG | down | 0.53 | -0.91 | 4.99E-02 |
